# Development and Validation of a Novel Tool for the Prediction of Clopidogrel Response in Chinese Acute Coronary Syndrome Patients: The GeneFA Score

**DOI:** 10.3389/fphar.2022.854867

**Published:** 2022-03-21

**Authors:** Hongyi Wu, Xiaoye Li, Juying Qian, Xin Zhao, Yao Yao, Qianzhou Lv, Junbo Ge

**Affiliations:** ^1^ Department of Cardiology, Zhongshan Hospital, Fudan University, Shanghai, China; ^2^ National Clinical Research Center for Interventional Medicine, Shanghai, China; ^3^ Department of Pharmacy, Zhongshan Hospital, Fudan University, Shanghai, China

**Keywords:** high platelet reactivity, genotype, clopidogrel, GeneFA score, ABCD-GENE score

## Abstract

**Aim:** Growing evidence indicated that CYP2C19 genotypes could only explain a fraction of the pharmacodynamic response to clopidogrel, while a number of clinical factors also have contributing roles. Our objective was to develop a new risk score to improve prognostication of ischemic events in Chinese patients treated with clopidogrel.

**Methods:** A new risk score was developed and internally validated in 445 patients with acute coronary syndrome (ACS) undergoing coronary stenting. The final score was named the GeneFA score based on the inclusion of CYP2C19 genotype, fibrinogen, and age. External validation of the GeneFA score and comparison with the ABCD-GENE score were performed in an independent ACS cohort.

**Results:** Based on the observed frequencies of high platelet reactivity (HRPR) in relation to the GeneFA risk score, a relatively higher clinical HRPR was observed in the upper quintile with a representative score of 3 (52.90%) and 4 (59.10%), whereas it was found less frequently in groups with scores 0 (6.70%), 1 (15.10%), and 2 (16.70%). Participants with a GeneFA score >2 had an increased risk of HRPR (54.3 *vs.* 14.7%, *p* < 0.001) and ischemic recurrence (20.7 *vs.* 5.4%, *p* < 0.001). The GeneFA score exhibited a better prediction for high HRPR patients as compared to the ABCD-GENE score (*p* < 0.001). In the validation population, GeneFA illustrated a similarly high prognostic value for HRPR incidence (C-statistic: 0.855 for GeneFA and 0.843 for ABCD-GENE) and ischemic recurrence (C-statistic: 0.726 for GeneFA and 0.724 for ABCD-GENE) on clopidogrel as compared to ABCD-GENE.

**Conclusion:** The GeneFA risk score had a moderate predictive ability for HRPR on clopidogrel for CAD patients in Chinese populations. The predictive value of the GeneFA score was consistent with the ABCD-GENE score for HRPR identification.

## Introduction

Platelet activation, which plays an important role in thrombosis formation, has been recognized as the cornerstone of secondary prevention in patients with coronary artery disease (CAD) (Tousoulis et al., 2010; Libby et al., 2019). Currently, dual antiplatelet therapy (DAPT) with aspirin and P2Y12 inhibitors is recommended as the standard treatment for patients with acute coronary syndrome (ACS), especially those undergoing percutaneous coronary intervention (PCI) operations (Valgimigli et al., 2018; Giacoppo et al., 2021). Although novel P2Y12 inhibitors such as ticagrelor and prasugrel have been shown to have greater potential inhibition of platelet aggregation (IPA), clopidogrel has remained widely used due to the benefits of relative lower costs, favorable safety and high compliance (Qutub et al., 2015). However, the unpredictability of clopidogrel’s efficacy poses a great challenge for clinical physicians. It was noteworthy that metabolism associated with genetic polymorphism might contribute to individual differences in the pharmacokinetics of clopidogrel, causing the variability in clopidogrel response (Pereira et al., 2019). Previous literature demonstrated that more than 20% of patients were in a prothrombotic state with high residual platelet reactivity (HRPR), which might lead to a high risk of ischemic events such as myocardial infarction and stent thrombosis after taking standard doses of clopidogrel (Di Nisio et al., 2005; Rafiq et al., 2017).

Currently, carriers of loss-of-function (LOF) alleles with the cytochrome P450 2C19 (CYP2C19) enzyme are recognized as a risk factor, causing an increased rates of HRPR and thrombotic complications (Wang et al., 2016; Biswas et al., 2021). Although the frequency of CYP2C19 LOF among Chinese is much higher than among Whites, there is no evidence to suggest Chinese have a poor response to clopidogrel. It might be explained by the various intrinsic platelet activities among different races (Jeong et al., 2011). In addition, CYP2C19 polymorphism leads to ethnic differences in the pharmacokinetics of clopidogrel. Therefore, it was excessive and inaccurate to guide P2Y12 inhibitor therapy only according to CYP2C19 genotype among Chinese patients with CAD. The previous literature indicated that a number of clinical factors such as age and fibrinogen, contributed to HRPR (Wu et al., 2020). Based on these results, clinical phenotype combined with CYP2C19 genetic testing may be more effective in identifying high-risk individuals.

Our objective was to develop a new risk score to improve the prognostication of ischemic events in Chinese ACS patients treated with clopidogrel.

## Methods

### Patient Population and Study Design

The risk score for predicting clopidogrel response was built from a study population that has been described previously (Wu et al., 2020). The trial was registered (URL: www.chictr.org, number: ChiCTR-OCH-11001767). Two patients without data on fibrinogen were excluded; a total of 445 patients with ACS undergoing coronary stenting were included in this analysis. External validation of the HRPR risk score was obtained by using an independent study population (Clopidogrel‐associated genetic variants on inhibition of platelet activity and clinical outcome for acute coronary syndrome patients), which focused on the pharmacodynamics and clinical outcomes of clopidogrel. The validation dataset enrolled 196 patients who were diagnosed with ACS.

All subjects received DAPT with the combination of aspirin (300 mg loading dose, 100 mg once daily) and clopidogrel (300 mg loading dose, 75 mg once daily). The Medical Ethics Committee of Zhongshan Hospital approved this study. Identified data were anonymized, and privacy issues were kept confidential.

The main exclusion criteria included the following: 1) receiving other antiplatelet agents or oral anticoagulants; 2) history of cerebral hemorrhage and known relevant hematological deviations; 3) discontinuation of clopidogrel antithrombotic; 4) lack of follow-ups.

### Baseline Demographics and Laboratory Measurements

Baseline demographics and laboratory measurements were collected through electronic medical records. The fibrinogen concentration measurement was performed by the following steps: 1) premix blood samples with thrombin solution, 2) deposit the mixture as droplets on a glass surface, and 3) allow the droplet to clot and apply a paper strip on top. The distance that wicks down the strip of blood was precisely related to the fibrinogen concentration.

An approximate venous blood sample (2 ml) for genotyping was collected into a vacutainer containing anticoagulant Ethylene Diamine Tetra‐acetic Acid (EDTA) from patients upon recruitment. Another whole peripheral blood sample (4 ml) was collected to measure the on-treatment platelet reactivity with thrombelastography (TEG; Haemoscope Corp, Niles, IL, United States) after receiving a loading dose of clopidogrel.

### Genotype Definitions

The DNA sample of each patient was isolated using the QIAamp DNA Blood Kit (Qiagen, Hilden, Germany) according to standard procedure. The single nucleotide polymorphisms (SNPs) including the CYP2C19 phenotype, were conducted by polymerase chain reaction (PCR) and TaqMan genotyping assays (Light Cycler 480, Roche, CA, United States). LOF alleles of CYP2C19*2 and *3 gene variants were the point mutations of 681G > A (rs4244285) and 636 G > A (rs4986893), respectively.

Individuals were classified into 3 genotype groups according to previous literature as extensive metabolizers (*1/*1), intermediate metabolizers (*1/*2 or *1/*3), and poor metabolizers (*2/*2, *2/*3 or *3/*3), respectively. We recorded the number of CYP2C19 LOF alleles as 0, 1, and 2 in extensive, intermediate, and poor metabolizers, respectively.

### Platelet Activity Assessment

We applied the Thrombelastograph Hemostasis Analyzer (Haemoscope Corp., Niles, Illinois, United States) with platelet mapping to measure platelet activity after medication with aspirin and clopidogrel. A blood sample (4 ml) was obtained for the TEG test, which is a point-of-care test to evaluate platelet and fibrin contributions to clot strength. The particular advantage of the TEG mapping system is that it evaluates antiplatelet effects after DAPT treatment simultaneously, including relative inhibition of platelet aggregation (IPA) and net residual (post-treatment) platelet activity. Adenosine diphosphate (ADP, 2 µmol/L 100 µL) induced maximal attitude (MA_ADP_) was considered as the residual platelet reactivity after clopidogrel treatment.

### GeneFA and ABCD-GENE Risk Score

The novel risk score is derived from the incorporation of 3 independent predictors on-clopidogrel HRPR, including 2 clinical and 1 genetic factor; it was named the GeneFA score based on the inclusion of CYP2C19 genotype, fibrinogen, and age. By analyzing the area under a combined receiver-operating characteristic (ROC) curve, age >60 years of age and fibrinogen value >310 mg/dl were identified as the optimal cut-offs. The total score was 4 and a cut-off score ≤2 represented high sensitivity and specificity to identify HRPR on clopidogrel in the proof-of-concept study. At present, the ABCD-GENE score was established to predict HRPR on clopidogrel among the European population based on 1 genetic and 4 clinical factors as follows: age >75 years (4 points), body mass index (BMI) > 30 kg/m^2^, chronic kidney disease (evaluated glomerular filtration rate <60 ml/min, 4 points), diabetes mellitus, and CYP2C19 LOF alleles. A cut-off score ≥10 was regarded as the best specificity and sensitivity for the identification of HRPR for clopidogrel (Angiolillo et al., 2020), as shown in Table 1.

**TABLE 1:** The characteristic of the GeneFA and ABCD-Gene risk score on HRPR.

GeneFA score*		Score	ABCD-gene score^#^		Score
Genotype testing					
CYP2C19 genotypes	None LOF carriers	0	CYP2C19 genotypes	None LOF carriers	0
	1 LOF carrier	1		1 LOF carrier	6
	2 LOF carriers	2		2 LOF carriers	24
Clinical factors					
Fibrinogen value > 310 (mg/dl)		1	Age > 75 (y)		4
			BMI > 30 (kg/m^2^)		4
Age > 60 (y)		1	CKD		3
			Diabetes		3

LOF, loss-of-function; BMI, body mass index; CKD, chronic kidney disease. CKD was indicated as estimated glomerular filtration rate <60 ml/min/1.73 m^2^.

The main objective of this study was to identify the diagnostic ability of the GeneFA score for HRPR on clopidogrel in the Chinese ACS population following PCI as compared to the ABCD-GENE score.

### Clinical Outcomes

The clinical outcome of the deviation population was major adverse cardiovascular events (MACE) during 12 months of follow-up through outpatient follow-ups and electronic medical record (EMR). The MACE was the composite of cardiovascular death, nonfatal myocardial infarction (MI), or stroke. The primary clinical endpoint of the validation dataset was MACE, including cardiovascular death, myocardial infarction, and revascularization for the targeted vascular lesion.

Cardiovascular death was regarded as any death with a demonstrable cardiovascular cause or any death not clearly attributable to a noncardiovascular cause. The diagnosis of MI is based on a new rise in troponin T ≥ 0.03 ng/ml associated with typical symptoms and/or typical electrocardiogram changes. The diagnosis of ischemic stroke requires clinical presentation and confirmation by computed tomography or magnetic resonance imaging of the head.

### Statistical Analysis

The descriptive statistics of continuous variables were expressed as means ± standard deviations (SD), and those of discrete variables were expressed as counts or percentages. Currently, the primary interest of this study was to test the specificity and sensitivity of the GeneFA score for prediction of HRPR on clopidogrel, which was defined as MA_ADP_ > 47.0 mm according to recent consensus statement (Tantry et al., 2013). In the developmental data set, a potential interaction of the risk score with MACE during follow-ups was evaluated, and the diagnostic ability of the GeneFA risk scoring system for MACE was tested in the validation data set.

One-way ANOVA was used to compare the differences of the continuous variables associated with HRPR, and a chi-squared test was performed to compare the distribution of categorical variables. We applied receiver-operating characteristic (ROC) curves to assess the discrimination performance of HRPR and ischemic events compared with GeneFA and ABCD-GENE scores. From the ROC curves, areas under the curve (AUCs) or c-statistics greater than 0.5 are considered to be of clinical value. Then, the GeneFA risk scoring system was performed to evaluate the discriminatory power of HRPR and ischemic events with the calculation of the area under the ROC in the validation data set.

Statistical analysis was performed using SPSS (IBM SPSS Statistics 22.0) and Prism 5 (GrandPad Software). A *p* value of 0.05 was considered as the threshold for statistical significance.

## Results

### Baseline Clinical Information in Developmental Datasets

Patients enrolled in the developmental datasets (N = 445) were representative of the previous study. Throughout 1 year of follow-up, a total of 38 patients sustained ischemic events, including 32 with nonfatal acute myocardial infarction, 3 experiencing nonfatal ischemic stroke, and 3 died in the developmental group. Meanwhile, a total of 102 (22.9%) participants were demonstrated to have HRPR (MA_ADP_ > 47.0 mm) based on results of platelet activity measurements. Variables including demographic information, comorbidity, laboratory measurements, concomitant medication, procedural characteristics, and LOF carriers of enrolled participants were collected as clinical and genotype factors.

As demonstrated in Table 2, the percentage of patients with an age >60 was 77.5% among subjects with HRPR and 57.7% among those without HRPR (*p* = 0.004). Similarly, 61.8% of patients with HRPR displayed high fibrinogen, whereas only 26.8% of patients without HRPR displayed high fibrinogen (*p* < 0.001). Moreover, the carriage of CYP2C19 LOF was more frequently observed in subjects with HRPR. After adjustment for confounding factors, these three parameters were independently and significantly associated with HRPR.

**TABLE 2:** Baseline characteristics of the development cohort.

Characteristics		HRPR			Multivariate analysis	
	Overall	No	Yes			
	N = 445	N = 343	N = 102	P	OR (95%CI)	P
Baseline characteristic						
Age > 60	277 (62.2%)	198 (57.7%)	79 (77.5%)	<0.001	2.292 (1.304, 4.028)	0.004
Male	356 (80.0%)	284 (82.8%)	72 (70.6%)	0.007	1.282 (0.619, 2.654)	0.504
BMI	24.8 ± 1.6	24.7 ± 1.6	24.9 ± 1.5	0.227		
Hypertension	287 (64.5%)	218 (63.6%)	69 (67.6%)	0.449		
Diabetes mellitus	133 (29.9%)	97 (28.3%)	36 (35.3%)	0.174	1.229 (0.731, 2.066)	0.438
Hypercholesterolemia	90 (20.2%)	66 (19.2%)	24 (23.5%)	0.344		
Smoking	264 (59.3%)	212 (61.8%)	52 (51.0%)	0.051	0.860 (0.455, 1.625)	0.642
Stroke	32 (7.2%)	23 (6.7%)	9 (8.8%)	0.467		
Previous PCI	61 (13.7%)	52 (15.2%)	9 (8.9%)	0.109	0.581 (0.256, 1.317)	0.193
Laboratory measurement						
Blood glucose (mmol/L)	6.2 ± 2.1	6.2 ± 2.1	6.2 ± 2.1	0.689		
White blood cell (x 10⁹/L)	7.3 ± 2.8	7.3 ± 2.8	7.2 ± 2.7	0.606		
Neutrophil (%)	64.4 ± 11.7	64.2 ± 11.9	64.8 ± 10.9	0.663		
Platelet count (x 10⁹/L)	194.3 ± 51.4	188.5 ± 48.9	210.8 ± 54.8	0.130		
Total cholesterol (mmol/L)	4.0 ± 1.0	4.0 ± 1.0	4.1 ± 0.9	0.264		
Triglyceride (mmol/L)	1.6 ± 1.1	1.6 ± 1.2	1.5 ± 0.8	0.295		
Low density lipoprotein (mmol/L)	2.2 ± 0.9	2.2 ± 0.9	2.3 ± 0.8	0.967		
Serum creatinine (umol/L)	78.3 ± 23.9	79.0 ± 25.7	75.6 ± 16.1	0.209		
Fibrinogen>310 mg/dl	155 (34.8%)	92 (26.8%)	63 (61.8%)	<0.001	4.365 (2.679, 7.112)	<0.001
Concomitant medication						
PPI	125 (28.1%)	89 (25.9%)	36 (35.3%)	0.065	1.284 (0.743, 2.221)	0.371
GPIIb/IIIa inhibitors	109 (24.5%)	79 (23.1%)	30 (29.4%)	0.194	1.275 (0.713, 2.280)	0.414
Coronary intervention procedure						
Targeted coronary artery				0.932		
LM	22 (4.9%)	16	6			
LAD	236 (53.0%)	184	52			
LCX	61 (13.7%)	46	15			
RCA	126 (28.3%)	97	29			
No. of stents	1.6 ± 0.8	1.6 ± 0.8	1.6 ± 0.8	0.732		
Length of stents (mm)	43.1 ± 24.1	43.3 ± 25.3	43.0 ± 23.0	0.909		
CYP2C19 LOF alleles				0.005		
No. of LOF carrier	175 (39.3%)	147	28			
1 LOF carriers	204 (45.8%)	153	51		1.902 (1.088, 3.325)	0.024
2 LOF carriers	66 (14.8%)	43	23		2.666 (1.310, 5.426)	0.007

Data are expressed mean ± SD or number of patients (percentage). BMI = body mass index; HRPR = high residual platelet reactivity; LAD = left anterior descending artery; LCX = left circumflex artery; LM = left main artery; LOF = loss-of-function; PPI = proton pump inhibitor; PCI = percutaneous coronary intervention; RCA = right coronary artery.

### Prevalence of HRPR for the Development Cohorts by Using the GeneFA Risk Score

On the basis of points assigned, patients were divided into five subgroups with a GeneFA score of 0–4 points. The different quintiles in absolute MA_ADP_ values are in the lower quintile (0–3 points): 17.8 (8.0–72.2) mm and in the upper quintile (3 and 4 points): 34.8 (12.6–83.2) mm.

**FIGURE 1:**
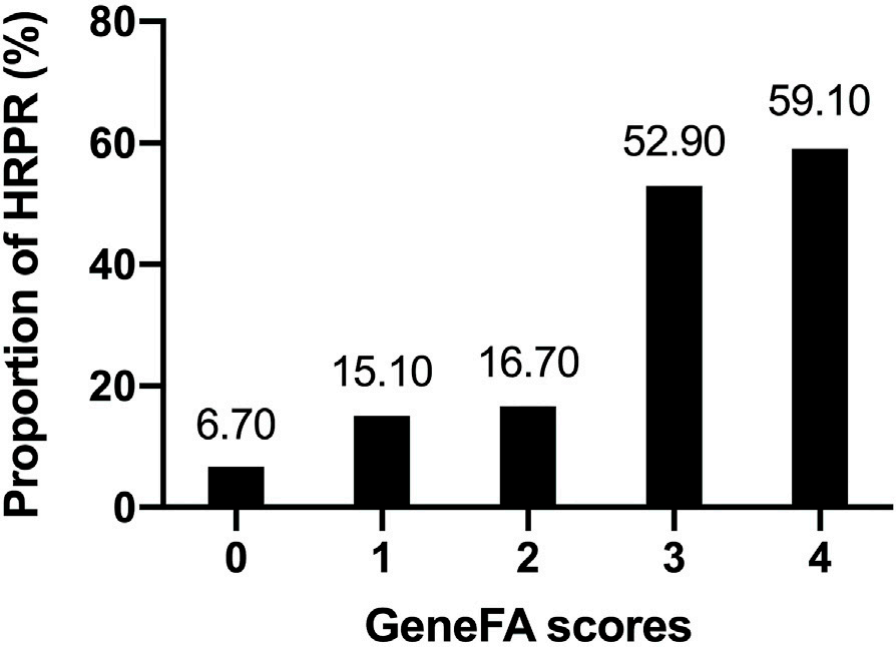
The prevalence of HRPR according to the GeneFA score in the development cohort.

Based on the observed frequencies of HRPR in relation to GeneFA risk score incorporation with clinical and genotype factors, the HRPR ratio in the development cohort was further presented according to each point (Figure 1). A relative higher clinically HRPR was observed in upper quintile with representative score of 3 (52.90%) and 4 (59.10%), meanwhile it was found less frequently in the score 0 (6.70%), 1 (15.10%), and 2 (16.70%) group, respectively.

### Clinical Implications of the GeneFA Risk Score for Prediction of HRPR and Ischemic Events

**FIGURE 2:**
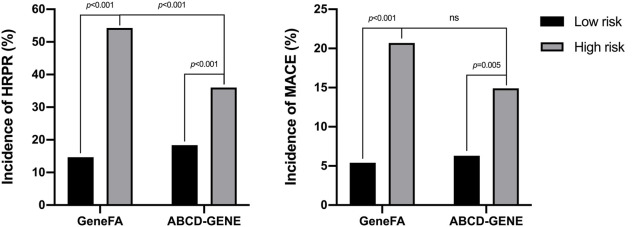
The incidence of HRPR on clopidogrel **(A)** and MACE **(B)** according to different risk scores derived from development data set.

Based on the cut-off risk scores of GeneFA >3 and ABCD-GENE ≥10, 445 participants were further categorized into low and high-risk groups (Figure 2A). When participants were divided into high and low risk groups according to the GeneFA score, participants with a GeneFA score >2 had an increased risk of HRPR (54.3 *vs.* 14.7%, *p* < 0.001) on clopidogrel (Figure 2A). Similarly, subjects with ABCD-GEN ≥10 also had an increased risk of HRPR (36.0 *vs.* 18.4%, *p* < 0.001). Moreover, the GeneFA score exhibited a better prediction for the presence of HRPR as compared to the ABCD-GENE score (*p* < 0.001).

On the basis of the cut-off, the GeneFA score significantly separated patients at higher and lower risk for MACE. As illustrated in Figure 2B, the high GeneFA score group exhibited a significantly higher ischemic rate compared to the low score group (20.7 *vs.* 5.4%, *p* < 0.001). The high ABCD-GENE score group displayed a significantly higher ischemic rate as compared with the low score group (14.9 *vs.* 6.3%, *p* = 0.005), and a similar modest ability was demonstrated to predict post-PCI ischemic events between the GeneFA and ABCD-GENE scores.

**FIGURE 3:**
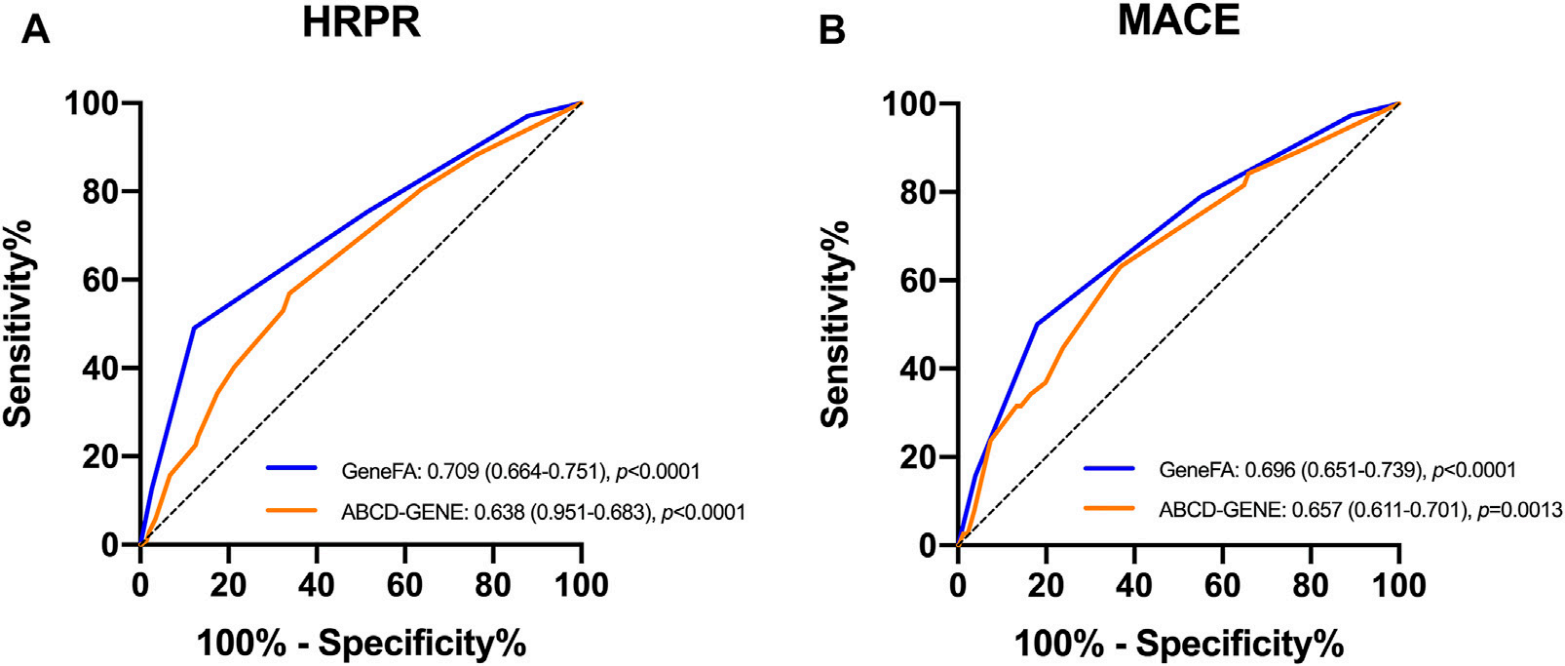
ROC determining model performance for the prediction of HRPR on clopidogrel **(A)** and MACE **(B)** in the developmental cohort; HRPR: high platelet reactivity.

The ROC curve showed that the GeneFA score had better discriminative power in predicting HRPR on clopidogrel in the development cohort as compared to ABCD-GENE score reflected by the AUC value (c-statistic: 0.709 for the GeneFA score and 0.638 for the ABCD-GENE score, respectively) (Figure 3A). Meanwhile, the sensitivity analysis illustrated that both the GeneFA and ABCD-GENE risk score seemed to have moderate predictive power in predicting MACE based on AUC value (c-statistic: 0.696 for GeneFA and 0.654 for the ABCD-GENE risk score, respectively) (Figure 3B).

### GeneFA Risk Score Analysis in the Validation Data Set

Then, we externally validated the developed prediction model in previous a clinical trial including 196 patients with 42 (21.4%) developing HRPR and 28 (14.3%) recent ischemic events during 1-year follow-up. External validation of each score was performed by GeneFA and ABCD-GENE. During the follow-up period, the HRPR on clopidogrel was significantly higher in high-risk groups as compared to low-risk groups according to the GeneFA risk score (65.6 and 12.8%, *p* = 0.001). Patients with a high GeneFA risk score were associated with a significantly higher rate of incidence of thrombosis, including hospitalization for revascularization, than patients with a low risk score (41.9 *vs.* 9.1%, *p* = 0.025).

**FIGURE 4:**
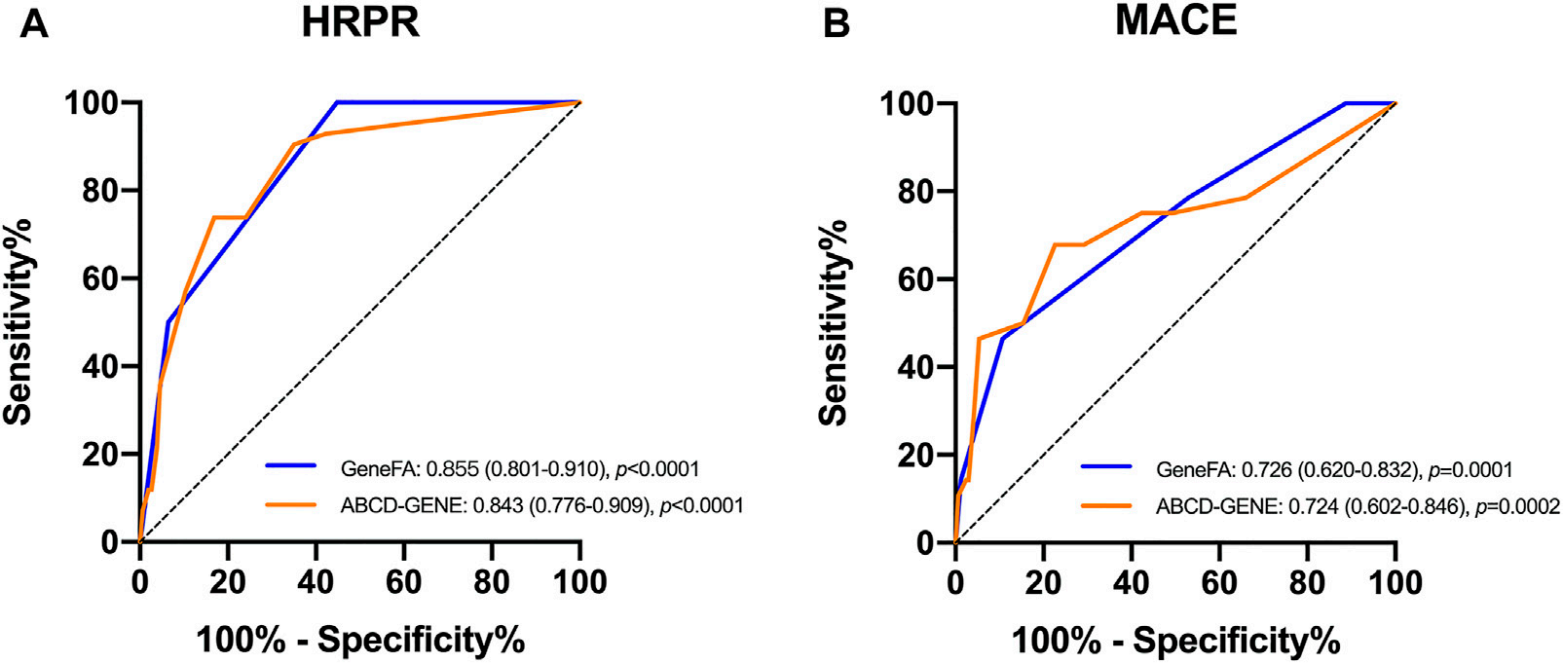
ROC determining model performance for the prediction of **(A)** HRPR incidence and **(B)** ischemic events on clopidogrel in the validation cohort.

We also compared the present risk score for predictive accuracy of HRPR incidence with the ABCD-GENE score in the validation population. The present new risk score had a similarly high prognostic value for HRPR incidence (C-statistic: 0.855 for GeneFA and 0.843 for ABCD-GENE) and ischemic recurrence (C-statistic: 0.726 for GeneFA and 0.724 for ABCD-GENE) on clopidogrel as compared to ABCD-GENE (Figure 4).

## Discussion

To the best of our knowledge, this was the first study to quantify the HRPR and ischemic risk of clopidogrel in patients with ACS using the GeneFA model. Thrombosis formation in patients with CAD remains life-threatening, with high morbidity and mortality (Bentzon et al., 2014). Platelet inhibitors are essential in the treatment and secondary prevention of patients with CAD following PCI (Kheiri et al., 2020). For several years, dual antiplatelet therapy remained the cornerstone of reduced major adverse cardiac events (Capodanno et al., 2018). Notably, about 10% of patients still experience recurring ischemic events during 1-year follow-up, despite current antiplatelet treatment (Shibata et al., 2015). Enhanced platelet reactivity is regarded as a risk factor for ischemic events in CAD patients (Dannenberg et al., 2020). Thus, abundant trials have been carried out to seek thrombosis biomarkers to predict clopidogrel response and individualize clopidogrel dosing regimens in clinical practice.

After screening and evaluating multiple factors, we demonstrated that age, fibrinogen, and genotypes of CYP2C19 were independently associated with HRPR on clopidogre in patients with ACS. According to many studies, growing age combined with preoperative coagulation function such as fibrinogen were described as independent risk factors of cardiovascular disease, mainly through compromised plasticity of vessels (North and Sinclair, 2012; Corella and Ordovás, 2014). Recently, elevated serum fibrinogen was demonstrated as an independent risk factor for ischemic events following PCI under clopidogrel treatment (Ang et al., 2017). The activated platelet surface receptors bind to circulating fibrinogen and fibrin molecules and facilitate platelet cross-linking, thrombosis, and clot formation (Rodrigues et al., 2006). As for the interaction between fibrinogen and platelet activation during thrombus formation, uncertainty remained regarding the independent effects of serum fibrinogen levels on adverse thrombosis and ischemic events (Griffiths et al., 2021). Our current study illustrated that an elevated serum fibrinogen level of 310 mg/dl is associated with significant platelet cross-linking and thrombus formation.

In accordance with previous clinical studies, CYP2C19*2 and *3 play important roles in the pharmacokinetic and pharmacodynamic effects of clopidogrel, and these LOF carriers were highly associated with higher platelet aggregation for patients treated with clopidogrel (Jiang et al., 2015; Pereira et al., 2019). In our study, CYP2C19 LOF alleles (*2 and *3) were related to HRPR for CAD patients with clopidogrel treatment. However, the impact of CYP2C19 LOF alleles on HRPR has been discussed in many studies and no firm conclusion has been drawn. Thus, further study is still needed to confirm the advantages of genotype-guided therapy for clopidogrel.

In our study, the GeneFA scoring system with risk factors of age, serum fibrinogen levels, and CYP2C19 LOF alleles was established to predict the HRPR for CAD patients with clopidogrel treatment in our study. Our results indicated that the GeneFA risk score could significantly predict HRPR on clopidogrel and the diagnostic ability of GeneFA score was consistent across a broad spectrum of East Asian populations with consistent cut-off values. Many published studies demonstrate that East Asian patients are prone to have a higher prevalence of HRPR and display a high risk profile of ischemic and bleeding events as compared to the Caucasian population (Saito et al., 2021). HRPR was mainly caused by inadequate platelet inhibition, which was frequently found in patients treated with clopidogrel (O’Connor et al., 2012). Totally, 73.2 and 68.0% of participants had HRPR on clopidogrel with the cut-off value of ADP-induced aggregation >47% in the developmental and validation cohorts of high GeneFA score. Due to the association of HRPR and ischemic events for patients following PCI operation, early detection of HRPR for thrombosis risk stratification and further potential intervention was of great importance. While previous published articles indicated that application of platelet function tests and genotype-guided antithrombotic strategies after PCI operation could decrease ischemic occurrence, it underscored the need for individualized antiplatelet treatment regimens in CAD patients (Hochholzer et al., 2014; Sibbing et al., 2019). Currently, the ABCD-GENE score, which combined clinical and genetic factors, was designed to stratify the antiplatelet response to clopidogrel. Despite the gene polymorphism influence on the conversion of the prodrug to its active metabolites for clopidogrel, clinical factors which also played an important role in leading to HRPR were also needed for consideration.

The ABCD-GENE risk score confirmed a proposition of clinical and genotype components to identify patients at HRPR on clopidogrel in different populations with age, BMI, CKD, diabetes, and CYP2C19 LOF alleles (Angiolillo et al., 2020). The predictive ability of the ABCD-GENE score in the developmental and validation cohorts was moderated by the AUC of ROC. Meanwhile, the GeneFA risk score included four risk factors and revealed quite good predictive ability for predicting HRPR on clopidogrel in CAD patients, validated in the developmental and validation cohorts in Chinese populations, respectively. Of note, the diagnostic ability and best cut-off values were both consistent between the ABCD-GENE and GeneFA risk scores. The consistency provides clinical evidence for the application of this new scoring system. Furthermore, the GeneFA risk score might be useful for management of clopidogrel response, contributing to the rapid performance of CYP2C19 genotype testing. However, both the ABCD-GENE and GeneFA risk scores had poor predictive value for clinical outcomes. The probable reason might be that many clinical factors could impact thrombosis formation in addition to HRPR.

### Conclusion

The GeneFA risk score had a moderate predictive ability for HRPR on clopidogrel for CAD patients in Chinese populations. The predictive value was consistent with the ABCD-GENE score, suggesting it to be clinically useful in HRPR identification.

### Limitations

There are several limitations to this study. First, some risk predictors in other scoring systems did not display statistical significance in the present study, which could be attributed to the limited sample size and short follow-up timeline. Therefore, a future large-scale clinical study is needed to confirm our hypothesis. Second, the study has the limitations inherent to the prospective cohort study, resulting in possible bias from selective prescription, and incidence of clinically relevant bleeding might be underestimated limited by the medical records. Third, items in some clinical outcomes could not be obtained owing to short follow-up periods, which might influence the score distribution and discriminative power. Thus, the results of this study need to be interpreted with caution in future clinical setting. Finally, we applied TEG as a measurement of platelet aggregation, which does not represent the functional gold standard for platelet function analysis. The HRPR for aspirin was not presented in this study, which caused the bias of MACE occurrence due to aspirin medication.

## Data Availability

The original contributions presented in the study are included in the article/Supplementary Material, further inquiries can be directed to the corresponding authors.
